# Effect of negotiation skills training program on head nurses’ knowledge and behavior

**DOI:** 10.1186/s12912-024-02581-w

**Published:** 2025-01-06

**Authors:** Hanaa M. Moustafa, Rabab M. Hassan, Fawzia M. Badran

**Affiliations:** Nursing Administration Department, Faculty of Nursing, Ain Shams University, Cairo, Egypt

**Keywords:** Negotiation, Head nurses, Knowledge, Behavior

## Abstract

**Background:**

Negotiating is a common occurrence and a significant part of everyday tasks for head nurses. The ability of the head nurse to effectively negotiate is a crucial management tool for work management in healthcare facilities.

**Aim:**

The present study aimed to assess the effect of negotiation skills training program on head nurses’ knowledge and behavior.

**Methods:**

A pretest–posttest, one-group quasi-experimental design was conducted at Menoufia University Hospitals. It is located in Shebeen, Elkom City, Cairo, Egypt. All head nurses and their assistants and their number 64 head nurses.

**Results:**

The results indicated that a minority of head nurses had satisfactory knowledge regarding negotiation in the pretest phase and obviously increased in the posttest to be more than three quarters and slightly decreased in the follow-up phase. Also, a minority had a high negotiation behavior level in the pretest phase and increased to be more than half in the posttest with a slight decrease in the follow-up stage. There was a highly statistically significant difference between pre-, post-, and follow-up results regarding negotiation knowledge and behavior.

**Conclusion:**

The study revealed that the head nurse’s knowledge and behavior increased markedly after implementing the negotiation skills training program.

**Recommendations:**

Conduct continuous education and training programs for stimulating and developing head nurses’ knowledge, behavior regarding negotiation, and hospital administration should support the importance of negotiation skills to both head nurses and organizations.

**Trial Registration Number [TRN]:**

The study protocol was approved by the Research Ethics Committee of the Faculty of Nursing, Ain Shams University (code number: NUR 23.10.131).

## Background

In the world of healthcare, negotiation is more than just a skill. It’s a fundamental administrative ability crucial to the success of any organization. Negotiation is defined as a strategic discussion intended to resolve an issue that both parties find acceptable. There are some cases that the supervisor faces in the work environment include workplace violence, shortage staff, long working hours, and workplace hazards [1].

Negotiation plays a key role in building strong relationships, delivering lasting and quality solutions, and avoiding short-term agreements that fail to satisfy all parties or lead to future conflicts. Effective negotiation requires a balance of give and take, with the head nurse striving to create mutually beneficial interactions that result in a win–win situation for everyone involved [2]. Ideally, a successful negotiation allows the nurse to make necessary concessions while still offering something valuable to the other party, benefiting both the staff and the organization. A well-conducted negotiation leaves all parties satisfied and ready to collaborate again in the future [3].

In the healthcare setting, the biggest challenge can reduce the negotiation is a conflict or absence of communication throughout the process, the agreement that is reached will not be as anticipated, and there is a strong chance that the outcome will only benefit one party without any challenge from the other side to arrive at a more equitable agreement, the absence of communication resulting from increasing the workload [4].

Communication makes human beings referred to as social creatures who cannot go off from the role of others. In addition, the ability to communicate effectively reflects one’s success, and a lack of it impedes the development of one’s personality. Communication skills can also be used to increase the effectiveness of professional skills, enhancement one’s confidence in the workplace when carrying out professional activities or interacting with colleagues to establish work relationships, among many other benefits [5].

In the negotiation, each party in a negotiation seeks to convince the other to support their position. A highly specialized form of human communication, negotiation is typically seen as a compromise to resolve a dispute or agreement in a way that will best serve the interests. The thread that will always be used to negotiate is communication. The dynamics of negotiation and communication are the same [6].

Head nurses are mediators, whether with staff nurses, patients, relatives, specialists, supervisors, or each other in negotiation process. Practical negotiation abilities have been recognized as a fundamental element of the head nurse role, and execution of the negotiation practice in today’s energetic as well as challenging wellbeing care setting is regularly hard, and tuning in to another’s point of view is noteworthy [7].

Ultimately, the head nurse’s role in negotiation as a first line manager in a working unit is in charge of prevention and resolving conflicts. Head nurses must acquire the negotiating skills required to obtain the resources needed for both themselves and their patients. In order to avoid confrontations with physicians, family members, patients, and other nurses must possess a broad range of understanding [8].

The head nurse is in charge of organizing, negotiating, and setting up a supportive work environment in order to prevent interdisciplinary conflicts. Also, can direct interdisciplinary conflicts in a constructive direction to deliver high-quality healthcare services. The head nurse is in charge of upholding a cooperative mindset, thinking well of other professions, accepting responsibility for any issues resolved, and ensuring that multidisciplinary staff members feel valued and supported [9].

In order to negotiate effectively, head nurses must understand and control the negotiation process at every stage before, during, and after the negotiation. This process can be divided into four key stages include preparation, opening, bargaining, and closure. Among these, the preparation stage is crucial, as it sets the foundation for the success of the entire negotiation [10].

During this stage, head nurses should gather as much relevant information as possible, including details about the other parties involved and any unique factors that may influence the negotiation. Being well-prepared boosts self-confidence and strengthens the mediator’s position [11]. The opening stage allows both parties to present their positions and conditions clearly. The bargaining stage involves negotiating toward an agreement, where influencing the other party is critical. Finally, the closure stage formalizes the agreement reached by both sides [12].

### Significance of the study

Negotiation plays a crucial role in the daily activities of head nurses, as most discussions that involve decision-making require both negotiation and time management skills. Head nurses regularly negotiate with patients, their families, nurses, managers, physicians, and other healthcare professionals to gain full cooperation and consent. Furthermore, negotiation helps define responsibilities, clarify ambiguities, and foster interpersonal and social relationships [13].

Negotiation relies on a set of skills that empower negotiators to excel in their roles. To address the complexities and challenges within an organization, it is crucial for negotiators to master these skills, gain awareness of various negotiation techniques, and understand the different concepts related to negotiation [14].

Role playing, case based learning are effective methods for improving the negotiation skills and attending periodical workshops, seminars, and training programs important for building the negotiation concept [15]. Therefore, this study aims to investigate the effect of negotiation skills training program on head nurses’ knowledge and behavior.

## Aim of the study

The present study aimed to assess the effect of negotiation skills training program on head nurses’ knowledge and behavior.

### Research hypothesis

Head nurses’ knowledge and behavior will be improved after attending negotiation skills training program.

## Methods

### Research design

Quasi-experimental design (pretest and posttest) one group was used to carry out this study.

### Research settings

This study was conducted at Menoufia University Hospitals. It locates in Shebeen – Elkom city, Cairo, Egypt. Its total bed capacity is (1000) beds, consists of 4 hospitals (emergency hospital, main hospital, specialized hospital, oncology hospital). These hospitals contain (19) critical units and (13) noncritical units**.**

### Sampling

The study subjects consisted of all head nurses and their assistants who are working at the previous mentioned settings. Their total number is (64) head nurses and their assistants. Convenience sample technique used in this study.

### Data measurements

Two tools were used for data collection, namely; Negotiation knowledge questionnaire, Negotiation self – assessment inventory.

### Tool 1: Negotiation knowledge questionnaire

This tool aimed to assess head nurses’ knowledge regarding negotiation and validity and reliability testing were conducted to ensure the robustness of the tool. It was consisted of 2 parts:—first part, Head nurse’s personal data and job characteristics including age, gender, marital status, qualifications, years of experience work department and attending workshops about negotiation. Second part, it was developed by the researcher guided by [10, 16–18]. This part included (30 questions) divided into two section. Section one, specific knowledge about negotiation was included (20 questions MCQ), (EX, Negotiation definition,importance of negotiation, principle of effective negotiation, preparing for successful negotiation), and section two general knowledge was included (10 questions true or false), (EX, A common mistake during the negotiation process is inaccurate information, the planning stage is the first step in the negotiation process).

A way to give points or marks to measure performance or achievements. The head nurse’s knowledge was tested with a model answer key. For each question, head nurses received a score of “1” for a correct answer and “0” for an incorrect answer. The smallest cutoff value is the minimum observed test value minus 1, and the largest cutoff value is the maximum observed test value plus 1. All the other cutoff values are the averages of two consecutive ordered observed test values. 18 is set as a cut-off point value. The scores were below 18 is considered unsatisfactory, while above 18 is considered satisfactory [19].

### Tool II: Negotiation self- assessment inventory

This tool aimed to assess head nurses behavior regarding negotiation. It was guided by [20] and validity and reliability testing were conducted to ensure the robustness of the tool. It was consisted of 25 statements (EX, If the other party’s position seems very important to him or her, I may sacrifice my own position, I address problems and concerns directly without blame or judgment, I try to win by convincing the other party of the logic and benefits of my position).

The answers from head nurses were rated on a 0–5-point scale are 0 = (never), 1 = rarely, 2 = sometimes, 3 = occasionally, 4 = frequently), and 5 = always.for each dimension, Mean score was calculated by subtracting the min value from max value and then dividing by number of levels to obtain equal width. A head nurse’s negotiation skills are considered high if the score is 92–125, moderate if the score is 59- 91 and 75 and low if the score is 25- 58 [20].

### Validity and reliability

The face and content validity of the studied scales were reviewed by a panel of experts consisting of seven experts from the nursing administration department affiliated with Ain Shams, Benha, and Menoufia Universities, Cairo, Egypt. The experts reviewed the measurements for completeness, clarity, significance, and thoughtful of applicability. The recommendations of the experts were careful, and necessary changes, amendments, and descriptions of the items were made accordingly. The reliability of the scales used in this study was assessed by Cronbach’s alpha coefficient test to determine the internal consistency of the study scales. The internal consistency reliability for the Negotiation Knowledge Questionnaire was 0.943, and that for the Negotiation Self- Assessment Inventory was 0.961.

### Ethical consideration

Before starting the study, the study plan was approved by the ethics committee at the Faculty of Nursing, Ain Shams University (code number: NUR 23.10.131). After getting permission from the nursing faculty at Ain Shams University. A letter explaining the purpose of the study was sent from the Dean of the Faculty of Nursing at Ain-Shams University to the nursing director of Menoufia University hospitals. We got the official approval to collect data and carry out the program. The researcher talked to the nursing directors at each hospital to explain what the study was about and how data would be collected during the training program. The researcher also asked for their approval and support for the study. Researcher introduced herself and explained the study’s purpose and goals. Also, showed them how to fill out the different forms and asked for their approval to take part and help with the study during the training program. Head nurses were told that any information shared would be kept private and only used for research. Head nurses were also informed that could leave the study at any time without needing to explain any reason.

### Operational design

**Fig. 1:**
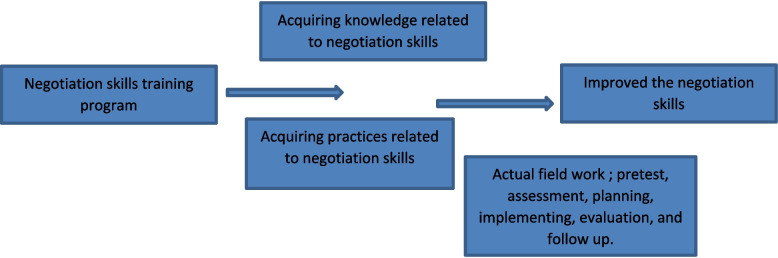
Conceptual model of the study

This design explains how to prepare the study tools (preparatory, conduct a pilot study, and carry out fieldwork) (Fig. 1).

### Preparatory phase

This phase began in May and ended in July 2023. In this stage, the researcher looked at books, journals, magazines, and theses from across the country and around the world related to different parts of the study. This helped create the tools for gathering data and plan the overall layout of the negotiation skills program.

### Pilot study

The pilot study was conducted on Six head nurses, who made up 10% of the main study group, took part in the pilot study. The pilot was used to check if the language was clear and if the tools could be used easily and effectively. It also helped to figure out how much time was needed to finish the data collection forms. The researcher needs to make changes. The pilot sample was excluded from the main study group.

### Field work

The study’s fieldwork began in June 2023 and will continue until the end of January 2024. It included steps assessment, planning, implementation, evaluation, and follow up.

#### Assessment phase

This phase lasted for one month in August 2023. After we finished creating the data collection tools using the results from the pilot study, we gathered information from the head nurses. Head nurses who agreed to take part in the study received the questionnaire forms at work before the program began. The researcher and hospital director will work together to set up the date and location for the program. A schedule was set for 2 days a week in the morning, with one session each day. The researcher was there to answer questions and prevent any confusion about the information. The researcher checked the sheets after finishing to make sure everything was complete. Each person took about 20 to 30 min to fill out the questionnaire. The same method was used right after the program and again three months later (check-in). The completed forms were given back to the researcher on the same day.

#### Planning phase

This phase lasted one month in September 2023. After finishing the data collection during the assessment phase, we analyzed the information. Using the information gathered from the assessment phase and reviewing related studies, the researcher created a training program to improve negotiation skills for head nurses. The main goal of the program was decided, a plan for the program was created, and the details of the program were outlined. We found ways to teach and reserved a place to hold the program sessions with the nursing department.

#### Implementation phase

This phase lasted two months, from October to November 2023. The researcher developing the training program after review of related literature, the researcher started the program with the head nurses. The program was scheduled to take 20 h. It included 10 sessions divided into (6 theoretical session, and 4 practical session) over 5 weeks, with 2 sessions each week. Each session lasted 2 h. In the first session, the researcher talked about what the training program is for, its goals, how it will be organized, managerial skills overview, second session talked about communication, while the third session (listening skills), fourth session (conflict), fifth session (conflict resolution strategies), sixth session (negotiation) seventh (negotiation process), eighth session(negotiation technique). Ninth session (emotional intelligence), finally the tenth session (critical thinking).

At the start of each session, we explained what the session was about and what we wanted to achieve. After each session, we talked informally about what was covered, how it was taught, and how well everyone understood. The researcher got the participants involved.

Different teaching methods were used in the program. These included small group discussions, role-playing exercises, case-based learning and real-life examples from work and everyday situations. The researcher developed a strong bond with the head nurses and encouraged them to take part in and share activities for the program.

Audio-visual tools like projectors were used. The researcher gave out a summary of the program’s sessions as a handout. This handout serves as a reminder and was shared with all head nurses, the nursing director, and the head of the nursing department.

#### Evaluation phase

This phase lasted one month in December 2023. After the program, a post-test was given to all the subjects using the same tools for collecting information that were used earlier. For the follow-up, we did the same process again three months after the evaluation at the end of the program, using the same tools to collect data. The researcher worked with the nursing department to gather information. A daily schedule was created each week based on how many subjects were involved in the work. The researcher’s phone number was put on each questionnaire so that people could easily ask questions. Each day, we collected around 8 to 10 sheets.

#### Follow up phase

This phase lasted one months through March 2023. By employing the same previous instruments, the follow-up test was conducted three months after the evaluation phase to examine the program’s impact again. In this phase, the data collection procedure was applied in the same way as in the assessment phase and post program evaluation phase (Table 1).

**Table 1:** Negotiation skills training program content

No	Session	Content
1	Managerial skills overview	Concept of managerial skills, Managerial skills, and importance of Managerial skills.
2	Communication overview	Concept of communication, importance of communication, barriers of communication, communication process, characteristics of good communicator, and challenges of communication
3	Listening skills overview	concept of active listening, importance of active listening, steps of listening process, Differentiation between hearing and listening, barriers of active listening, listening techniques, and how to improve active listening
4	Conflict overview	concept of conflict, causes of conflict—and consequences of conflict
5	Conflict resolution strategies	Concept of conflict resolution strategies, sources of organizational conflict, and conflict process
6	Concept of negotiation	concept of negotiation, importance of negotiation, characteristics of good negotiator, negotiation types, and nurse manager role as a negotiator
7	Negotiation process	concept negotiation process, steps of negotiation, barriers to successful negotiation, steps of negotiation, and negotiation outcomes
8	Negotiation techniques	Concept of negotiation techniques, importance of negotiation in healthcare settings, key skills required for successful negotiation, principles of negotiation, and factor a affecting negotiation
9	Emotional intelligence overview	concept of emotional intelligence, importance of emotional intelligence, characteristics of emotionally intelligent person, dimensions of emotional intelligence, and strategies to improve emotional intelligence
10	Critical thinking overview	Concept of thinking, critical thinking, critical thinking skills -, importance of critical thinking, characteristics of critical thinker -, critical thinking process, critical thinking skills, barriers to critical thinking, and strategies to promote critical thinking

### Statistical design

Data entry and statistical analysis were done using SPSS 24 statistical software package. It helps us figure out the best way to collect information so we can understand and analyze it better. The data was shown as counts and percentages for categories, and as averages and standard deviations for numbers. We used a Chi-square test to compare different categories. A paired t-test was used to compare the averages of the same group before and after the treatment, as well as to compare the averages after the treatment and during the follow-up period. The *P* value is important if it is 0. 05 or lower.

## Results

### Frequency distribution of head nurses’ personal and work-related data (*n* = 64)

Table 2 Shows that the majority (82.8%) of head nurses were > 40 years old, more than three quarters (78.1%) of them had BSc, the majority (85.9%) of them were married, the great majority (92.2%) of them had > 20 years of experience, more than half (57.8%) of them were working in inpatient units, and nobody of them had attended any workshop about negotiation skills.

**Table 2:** Frequency distribution of staff nurses’ personal and work-related data (*n* = 64)

Personal and work-related data	No	%
**Age:**		
30–40 years	11	17.2
> 40 years	53	82.8
Mean ± SD	42.14 ± 3.09	
**Qualification:**		
BSc	50	78.1
Institute	14	21.9
**Marital status:**		
Married	55	85.9
Single	9	14.1
**Experience:**		
< 20 years	5	7.8
20 years and more	59	92.2
**Work department:**		
Critical areas	27	42.2
Inpatient	37	57.8
**Attending work shop about negotiation skills:** No	64	100

### Head nurses’ satisfactory knowledge regarding negotiation throughout program phases (*n* = 64)

Tables 3 and 4 Reveals that there were highly significant statistical differences (*p* = 0.00) in head nurses’ knowledge items with elevation in posttest and follow up than pretest, more than three quarters of head nurses had unsatisfactory knowledge regarding negotiation in pretest, however, in posttest and follow up stage head nurses developed satisfactory knowledge regarding negotiation, majority of head nurses had unsatisfactory level regarding importance of negotiation in pretest stage, while, it improved in posttest and follow up during the program.

**Table 3:** Head nurses’ satisfactory knowledge regarding negotiation throughout program phases (*n* = 64)

Knowledge	Pretest				Posttest				Follow up				Pre-post		Pre-F.up	
	**Unsatisfactory**		**satisfactory**		**Unsatisfactory**		**satisfactory**		**unsatisfactory**		**satisfactory**					
	**No**	**%**	**No**	**%**	**No**	**%**	**No**	**%**	**No**	**%**	**No**	**%**	**χ2**	***P***	**χ2**	***P***
Communication definition	55	85.9	9	14.1	13	20.3	51	79.7	16	25.0	48	75.0	124.1	.00	120.8	.00
Conflict definition	50	78.1	14	21.9	12	18.8	52	81.3	17	26.6	47	73.4	124.1	.00	122.1	.00
negotiation definition	52	81.3	12	18.8	15	23.4	49	76.6	15	23.4	49	76.6	128.0	.00	111.9	.00
aspects of assertiveness	49	76.6	15	23.4	17	26.6	47	73.4	20	31.3	44	68.8	120.2	.00	109.4	.00
value creation in negotiation helps	43	67.2	21	32.8	14	21.9	50	78.1	19	29.7	45	70.3	120.2	.00	120.2	.00
importance of negotiating techniques	52	81.3	12	18.8	12	18.8	52	81.3	19	29.7	45	70.3	124.1	.00	112.8	.00
characteristic of negotiation	52	81.3	12	18.8	16	25.0	48	75.0	22	34.4	42	65.6	128.0	.00	110.0	.00
importance of negotiation	59	92.2	5	7.8	14	21.9	50	78.1	21	32.8	43	67.2	96.4	.00	91.8	.00
principle of effective negotiation	51	79.7	13	20.3	13	20.3	51	79.7	19	29.7	45	70.3	124.1	.00	89.6	.00
preparing for successful negotiation	48	75.0	16	25.0	17	26.6	47	73.4	25	39.1	39	60.9	99.6	.00	85.8	.00
the environmental barrier of negotiation	49	76.6	15	23.4	14	21.9	50	78.1	23	35.9	41	64.1	93.4	.00	79.3	.00
irrelevant principle of successful negotiation	46	71.9	18	28.1	13	20.3	51	79.7	20	31.3	44	68.8	120.2	.00	110.5	.00
stages of negotiation	44	68.8	20	31.3	18	28.1	46	71.9	27	42.2	37	57.8	96.4	.00	90.2	.00
aim of negotiation process	47	73.4	17	26.6	12	18.8	52	81.3	17	26.6	47	73.4	90.4	.00	99.4	.00
successful point for negotiation	53	82.8	11	17.2	11	17.2	53	82.8	18	28.1	46	71.9	90.4	.00	94.6	.00
qualities of negotiator	54	84.4	10	15.6	14	21.9	50	78.1	19	29.7	45	70.3	93.4	.00	91.7	.00
integrative negotiation	47	73.4	17	26.6	13	20.3	51	79.7	16	25.0	48	75.0	11.1	.00	19.4	.00
negotiation style for ensuring a win–win outcome	52	81.3	12	18.8	15	23.4	49	76.6	19	29.7	45	70.3	87.6	.00	80.6	.00
a common negotiation mistake	45	70.3	19	29.7	13	20.3	51	79.7	18	28.1	46	71.9	93.4	.00	93.2	.00
unsuccessful strategy for negotiation	48	75.0	16	25.0	17	26.6	47	73.4	25	39.1	39	60.9	105.0	.00	110.6	.00

**Table 4:** Head nurses’ satisfactory knowledge regarding negotiation throughout program phases (*n* = 64)

Knowledge	Pretest				Posttest				Follow up				Pre-post		Pre-F.up	
	**Unsatisfactory**		**satisfactory**		**Unsatisfactory**		**Satisfactory**		**unsatisfactory**		**satisfactory**					
	**No**	**%**	**No**	**%**	**No**	**%**	**No**	**%**	**No**	**%**	**No**	**%**	**χ2**	***P***	**χ2**	***P***
A successful negotiator can start the negotiation process without prior defining the objectives of the negotiation process	51	79.7	13	20.3	16	25.0	48	75.0	24	37.5	40	62.5	52.6	.00	56.9	.00
It is necessary, to eliminate the success of negotiation, compare the results of the negotiations with the objectives reached.	53	82.8	11	17.2	12	18.8	52	81.3	19	29.7	45	70.3	90.4	.00	92.1	.00
Tact and persuasion are skills of good negotiator should have gained.	47	73.4	17	26.6	19	29.7	45	70.3	24	37.5	40	62.5	74.3	.00	70.2	.00
The ability to change facts is a prerequisite for successful persuasion.	46	71.9	18	28.1	11	17.2	53	82.8	16	25.0	48	75.0	99.6	.00	90.1	.00
A common mistake during the negotiation process is inaccurate information.	45	70.3	19	29.7	14	21.9	50	78.1	18	28.1	46	71.9	96.4	.00	76.4	.00
The planning stage is the first step in the negotiation process.	54	84.4	10	15.6	13	20.3	51	79.7	18	28.1	46	71.9	93.4	.00	33.4	.00
It is important, during the negotiation planning phase, to visualize other available alternatives.	47	73.4	17	26.6	12	18.8	52	81.3	19	29.7	45	70.3	74.3	.00	35.3	.00
Before starting negotiation, it is necessary to gather data about other party weakness and strength.	51	79.7	13	20.3	15	23.4	49	76.6	17	26.6	47	73.4	84.8	.00	97.8	.00
You should only talk about your goals when trying to build a relation with other party.	44	68.8	20	31.3	12	18.8	52	81.3	13	20.3	51	79.7	93.4	.00	80.3	.00
Discussing positively with other party create negative environment	41	64.1	23	35.9	14	21.9	50	78.1	16	25.0	48	75.0	36.0	.00	46.3	.00

^*^P is significant at ≤ 0.05

### Head nurses’ negotiation behavior level throughout program phases (*n* = 64)

Tables 5 and 6 Displays that there were highly significant statistical differences (*p* = 0.00) in head nurses’ negotiation behavior items in posttest, follow up and pretest, head nurses had marked increase in head nurse’s negotiation behavior at posttest and follow up phase when compared with pretest phase.

**Table 5:** Head nurses’ negotiation behavior level throughout program phases (*n* = 64)

Negotiation behavior	Pretest						Posttest						Follow up						Pre-post		Pre-F.up	
	**Low**		**Moderate**		**High**		**Low**		**Moderate**		**High**		**Low**		**Moderate**		**High**					
	**No**	**%**	**No**	**%**	**No**	**%**	**No**	**%**	**No**	**%**	**No**	**%**	**No**	**%**	**No**	**%**	**No**	**%**	**χ2**	***P***	**χ2**	***P***
If the other party’s position seems very important to him or her, I may sacrifice my own position.	40	62.5	17	26.6	7	10.9	12	18.8	8	12.5	44	68.8	15	23.4	11	17.2	38	59.4	7.09	.00	8.19	.00
I address problems and concerns directly without blame or judgment	36	56.3	19	29.7	9	14.1	11	17.2	15	23.4	38	59.4	12	18.8	16	25.0	36	56.3	6.12	.00	7.23	.00
I try to win by convincing the other party of the logic and benefits of my position.	29	45.3	30	46.9	5	7.8	14	21.9	15	23.4	35	54.7	15	23.4	15	23.4	34	53.1	12.1	.00	16.9	.00
I tell the other person my ideas for and ask for his or hers in return	33	51.6	25	39.1	6	9.4	9	14.1	23	35.9	32	50.0	11	17.2	23	35.9	30	46.9	10.9	.00	14.3	.00
I try to find a compromise solution.	36	56.3	16	25.0	12	18.8	7	10.9	28	43.8	29	45.3	7	10.9	30	46.9	27	42.2	9.00	.00	7.09	.00
I try to postpone discussions until I have had some time to think.	28	43.8	25	39.1	11	17.2	10	15.6	21	32.8	33	51.6	13	20.3	22	34.4	29	45.3	12.0	.00	14.3	.00
I see achievement as more important than relational issues.	30	46.9	24	37.5	10	15.6	11	17.2	22	34.4	31	48.4	14	21.9	23	35.9	27	42.2	11.4	.00	15.3	.00
I use body language that might be perceived as condescending or arrogant.	31	48.4	18	28.1	15	23.4	9	14.1	19	29.7	36	56.3	14	21.9	22	34.4	28	43.8	7.80	.00	6.89	.00
Confronting someone about a problem is very uncomfortable for me.	27	42.2	23	35.9	14	21.9	7	10.9	19	29.7	38	59.4	8	12.5	21	32.8	35	54.7	8.92	.00	7.90	.00
I give up some points in exchange for others.	26	40.6	25	39.1	13	20.3	7	10.9	26	40.6	31	48.4	8	12.5	29	45.3	27	42.2	6.34	.00	9.80	.00
I propose a middle ground.	39	60.9	9	14.1	16	25.0	9	14.1	25	39.1	30	46.9	10	15.6	26	40.6	28	43.8	5.35	.00	4.32	.00
I am likely to take a comment back or try to soften it if I realize that it hurt someone’s feelings.	37	57.8	10	15.6	17	26.6	11	17.2	15	23.4	38	59.4	12	18.8	16	25.0	36	56.3	6.09	.00	8.19	.00
I think it is all right to ask for what I want or to explain how I feel.	29	45.3	25	39.1	10	15.6	13	20.3	15	23.4	36	56.3	15	23.4	19	29.7	30	46.9	4.32	.00	3.02	.00
I find conflict stressful and will avoid it any way I can.	32	50.0	21	32.8	11	17.2	12	18.8	15	23.4	37	57.8	13	20.3	18	28.1	33	51.6	15.70	.00	11.2	.00

Table 5 represents the Head nurses’ total negotiation behavior mean scores throughout program phases, that's mean level of negotiation improved

**Table 6:** Head nurses’ negotiation behavior level throughout program phases (*n* = 64)

Negotiation behavior	Pretest						Posttest						Follow up						Pre-post		Pre-F.up	
	**Low**		**Moderate**		**High**		**Low**		**Moderate**		**High**		**Low**		**Moderate**		**High**					
	**No**	**%**	**No**	**%**	**No**	**%**	**No**	**%**	**No**	**%**	**No**	**%**	**No**	**%**	**No**	**%**	**No**	**%**	**χ2**	***P***	**χ2**	***P***
1. I have been described as impatient, controlling, insensitive or emotionally detached	33	51.6	17	26.6	14	21.9	14	21.9	18	28.1	32	50.0	15	23.4	18	28.1	31	48.4	5.45	.00	4.46	.00
2. If asked to do something I don’t agree with or don’t want to do, I’ll do it but deliberately won’t do it as well as I could have.	35	54.7	17	26.6	12	18.8	11	17.2	22	34.4	31	48.4	16	25.0	21	32.8	27	42.2	7.00	.00	8.25	.00
3. I let my body language communicate my feelings rather than telling people directly how I feel.	35	54.7	14	21.9	15	23.4	14	21.9	20	31.3	30	46.9	14	21.9	21	32.8	29	45.3	3.21	.00	2.09	.00
4. I remain calm and confident when faced with aggression or criticism.	36	56.3	15	23.4	13	20.3	9	14.1	21	32.8	34	53.1	12	18.8	21	32.8	31	48.4	3.78	.00	5.12	.00
5. I may overextend myself trying to meet everyone’s needs.	37	57.8	15	23.4	12	18.8	7	10.9	17	26.6	40	62.5	10	15.6	18	28.1	36	56.3	5.80	.00	8.02	.00
6. I try to find fair combination of gains and losses for both of us	31	48.4	23	35.9	10	15.6	6	9.4	17	26.6	41	64.1	9	14.1	16	25.0	39	60.9	7.32	.00	6.21	.00
7. I look for and acknowledge common ground	32	50.0	19	29.7	13	20.3	4	6.3	15	23.4	45	70.3	8	12.5	16	25.0	40	62.5	5.08	.00	5.00	.00
8. I have a hard time being clear about what I want and need for fear of appearing demanding or selfish	34	53.1	23	35.9	7	10.9	12	18.8	10	15.6	42	65.6	13	20.3	13	20.3	38	59.4	3.26	.00	2.12	.00
9. I can overlook valuable ideas in favor of action	31	48.4	25	39.1	8	12.5	5	7.8	28	43.8	31	48.4	7	10.9	26	40.6	31	48.4	7.32	.00	5.09	.00
10. I may not be open to hear other points of view.	37	57.8	17	26.6	10	15.6	6	9.4	25	39.1	33	51.6	6	9.4	25	39.1	33	51.6	1.09	.00	.99	.00
11. I avoid taking positions that would create controversy	38	59.4	13	20.3	13	20.3	9	14.1	25	39.1	30	46.9	10	15.6	26	40.6	28	43.8	7.00	.00	4.20	.00

^*^P is significant at ≤ 0.05

### Head nurses’ total negotiation behavior mean scores throughout program phases

Table 7 Shows that there were highly significant statistical differences (*p* = 0.00) in head nurses’ total negotiation behavior mean scores with elevation in posttest (x̅ ± SD = 92.15 ± 23.09) and follow up (x̅ ± SD = 85.43 ± 24.05) than pretest (x̅ ± SD = 40.17 ± 10.6).

**Table 7:** Head nurses’ total negotiation behavior mean scores throughout program phases

Negotiation behavior	Pretest	Posttest	Follow up	Pre-post		Pre-F. up	
	**Mean ± SD**	**Mean ± SD**	**Mean ± SD**	**T**	***P***	**T**	***P***
**Total negotiation behavior**	40.17 ± 10.6	92.15 ± 23.09	85.43 ± 24.05	136.78	.00	121.4	.00

^*^P is significant at ≤ 0.05

### Correlation between negotiation knowledge, negotiation behavior, among head nurses

Table 8 Concludes that there were significant statistical positive correlations (*r* = 0.82, *p* = 0.00) between negotiation knowledge, and negotiation behavior among head nurses.

**Table 8:** Correlation between negotiation knowledge, negotiation behavior, among head nurses

Pretest Spearman rank correlation		Total knowledge
**Total negotiation behavior**	R	0.82
	*P*	0.00^*^

^*^P is significant at ≤ 0.05

## Results

### Discussion

Negotiation is a social process that is seen as a key skill in many areas of work especially in healthcare facilities. It helps people make agreements, settle arguments, share resources, make choices, and fix problems. Negotiation is a way to solve problems or arguments between two or more people. Head nurses share ideas and responses to communicate with each other. Each side tries hard to get the best results. Negotiations happen every day about many topics [1].

Negotiation relies on skills that help people be successful in their roles. That’s why head nurses need to learn these skills and understand how to resolve problems and challenges that come up in a group. Head nurses should also be aware of different negotiation methods and ideas. Human skills refer to head nurses’ ability to understand what motivates their team and use the right leadership skills to reach the hospital’s goals and provide a good service to the patient. Good leadership skills include the head nurse’s ability to negotiate. The skills of the head nurse can greatly affect how negotiations go [21].

Concerning head nurses’ negotiation knowledge, the result of the present study revealed that more than three-quarters of head nurses had unsatisfactory knowledge regarding negotiation in the pretest; however, in the posttest and follow-up stages, head nurses developed satisfactory knowledge regarding negotiation. From the researcher’s point of view, this may be due to the fact that this result can be attributed to the comprehensive nature of the training and reinforcement mechanisms in the program. This finding was in line with [22], who reported that the majority of nurses acquired knowledge related to negotiation immediately after program implementation.

Regarding negotiation knowledge, the results of the current study are consistent with a study conducted by [23] who reported a positive reaction and increased knowledge among participants after exposure to a soft skills training program including negotiation. Similarly, in line with these findings, a study by [24] who identified highly statistically significant differences in nurses’ knowledge immediately post-intervention compared to the pre-program period. Furthermore, the present study aligns with the research conducted by [25], as they observed a highly significant difference in knowledge level scores before and after the implementation of a training program. From the researcher’s perspective, this improvement in knowledge may be attributed to the head nurses’ necessity to acquire additional information about various skills utilized in unit management. Such knowledge is deemed crucial for enhancing their efficiency in clinical practice and contributing to their career advancement.

Concerning negotiation knowledge, the study result revealed that the majority of head nurses had an unsatisfactory level regarding the importance of negotiation in the pretest stage, while it improved in the posttest and follow-up during the program. This may be related to the lack of interest of head nurses and the work environment that enhances the negotiation process in a health care setting. This finding is consistent with the study of [26], who revealed that success of the negotiation process in any health care setting depends mainly on understanding the importance of negotiation, the need for negotiation, and the successful points for effective negotiation to achieve the desired goals.

The present study demonstrated a marked increase in the head nurse’s negotiation behavior at the posttest and follow-up phases when compared with the pretest phase. From the researcher’s point of view, this may be due to incorporating role-play exercises, providing feedback, encouraging active listening, and emphasizing win–win situations. The result is in the same line with [21], who emphasized that any behavior needs time to be noticed and implemented, as the ability to negotiate is important to nurses because negotiation skills develop critical thinking and effective communication skills that are important in healthcare settings and enhance the negotiation process.

The findings of the present study demonstrated that the total negotiation behavior mean score of head nurses throughout the program phases was obviously increased in the post-program phase. There were highly statistically significant differences in negotiation behavior among head nurses throughout the program phases. This result is supported by [27], who clarified that there was a general improvement in head nurses’ negotiation knowledge at post-program and follow-up as compared to the pre-program stage. Knowledge improvement will elevate negotiation behavior.

Regarding total negotiation behavior score, the current study’s results align with the findings of [23], who reported increased mean scores across all participants’ skills after a four-month training. Additionally, these results are consistent with a study by [15], which demonstrated the beneficial effects of a program on nursing students’ communication, clinical interaction skills, and positive problem-solving, conflict resolution and negotiation. From the researcher’s perspective, this result can be attributed to the education program raising awareness among head nurses.

Finally, our present study concluded that there were significant statistically positive correlations between negotiation knowledge and negotiation behavior among head nurses. This was consistent with [28], who concluded that employees who attended a program titled “The Impact of Training Sessions on Physical Security Awareness: Measuring Employees’ Knowledge, Attitude, and Self-Reported Behavior) had developed a noticeable change in behavior. It was found that there was a significant relationship between employees’ knowledge and attitude and their self-reported behavior after attending training sessions.

Regarding correlation between knowledge and behavior, The findings of the current study are consistent with the study conducted by [29], which demonstrated strong empirical support for the causal relationship between knowledge acquired by employees and their work performance, also demonstrated a significant positive correlations between knowledge, and practice among head nurses immediately post program implementation and post three months later(follow-up). From the researcher’s perspective, this improvement could be attributed to the implementation of the training program and the acquisition of various knowledge and skills by head nurses throughout the program concerning negotiation skills and their practical application in the workplace.

## Conclusion

The study found that the training program helped head nurses improve their knowledge and behavior. Before the training, more than three-quarters of the head nurses did not know much about negotiation. However, after the training and follow-up, they gained a good understanding of negotiation. Initially, most head nurses also did not see the importance of negotiation, but their views improved after the training. Overall, the head nurses showed a significant increase in their knowledge and behavior related to negotiation after the program.

### Implications of the study

The study’s findings indicate that targeted training programs are effective in enhancing head nurses’ knowledge and behavior regarding negotiation. The significant improvements in both their understanding and attitudes toward negotiation emphasize the value of integrating such training into the ongoing professional development of nursing leaders. This suggests that similar programs could play a crucial role in closing knowledge gaps and shifting attitudes among healthcare professionals, thereby enhancing their ability to manage conflicts and communicate more effectively in the workplace.

Additionally, the observed positive change in head nurses’ views on the importance of negotiation suggests that these programs can promote a culture of continuous learning and professional development, which is vital for the ever-evolving healthcare environment. Expanding this training to include a wider range of nursing staff could result in more cohesive teamwork, improved decision-making, and better patient care outcomes. This research highlights the importance for healthcare organizations to prioritize negotiation skills training as a key element of leadership development and quality improvement efforts.

## Data Availability

Due to confidentiality concerns, the data and materials used in the current research cannot be made publicly available. However, they are available from the corresponding author upon reasonable request.
